# Supplementary material for: Predicting optimal deep brain stimulation parameters for Parkinson’s disease using functional MRI and machine learning
Source: Nat Commun. 2021 May 24;12:3043. doi: 10.1038/s41467-021-23311-9 (PMC8144408; doi:10.1038/s41467-021-23311-9)
Supplement: Supplementary file 1 — Supplementary Information [file 41467_2021_23311_MOESM1_ESM.pdf]

Functional MRI-based Individualized Programming of
Deep Brain Stimulation for Parkinson's disease

Supplementary Information

	Study	Target	Number of Patients	Field Strength (tesla)	Stimulation Mode	Analysis	Main Findings
Prospective	Horn et al. (2019)[1]	STN	20	1.5	Bipolar	Brain connectivity changes with stimulation	-Increasing STN stimulation with effective stimulation increased connectivity in the motor network<br>-Effective stimulation restores connectivity
	Gratwicke et al. (2018)[2]	NBM	6	1.5	N/S	Acute stimulation effects	No change in default mode network
	Mueller et al. (2018)[3]<br>Holiga et al. (2015)[4]<br>Mueller et al. (2013)[5]	STN	13	1.5	Bipolar	Brain connectivity changes with stimulation	-Increased connectivity between motor cortex, thalamus, cerebellum<br>-Increased connectivity in premotor cortex
	Saenger et al. (2017)[6]<br>Kahan et al. (2014)[7]<br>Kahan et al. (2012)[8]	STN	12	1.5	N/S	Brain connectivity changes with stimulation	-Effective stimulation restores connectivity<br>-Modulatory effects of stimulation best described with direct, indirect, and hyperdirect pathways
	Knight et al. (2015)[9]	STN	10	1.5	Bipolar	Acute stimulation effects	Motor circuit engagement

	Jech et al. (2012)[10]	STN	12	1.5	None	Microlesioning effect on movements	-Microlesioning effect lowered movement-related cortical and subcortical activation
	Phillips et al. (2006)[11]	STN	5	3	N/S	Acute stimulation effects	Motor circuit engagement
	Hesselman et al. (2004)[12]	STN	1	1.5	Bipolar	Acute stimulation effects	Motor circuit engagement
	Stefurak et al. (2003)[13]	STN	1	1.5	Bipolar	Acute stimulation effects	Motor circuit engagement
	Jech et al. (2001)[14]	STN, VIM	4	1.5	Bipolar	Acute stimulation effects	Motor circuit engagement
Retrospective	De Almeida et al. (2019)[15]	STN	20	N/A	N/A	Brain connectivity associated with short-term motor leaning	Short-term motor learning was associated with motor circuit engagement, particularly the cerebellum
	Horn et al. (2017)[16]	STN	95	N/A	N/A	Brain connectivity associated with clinical benefits	Clinical benefits were associated with motor circuit engagement

Supplementary Table 1: fMRI studies in Parkinson's disease patients with DBS. Prospective and retrospective fMRI studies in Parkinson's disease patients with DBS. Prospective studies refer to acquisition of fMRI with the DBS leads implanted (with or without stimulation) whereas retrospective refers to computing functional connectivity associated with stimulation location, most commonly

with healthy normative resting-state fMRI. N/A = not applicable; NBM = nucleus basalis of Meynert; N/S = not specified; STN = subthalamic nucleus; VIM = ventral intermediate nucleus.

	TR ms	TE ms	TI ms	BW kHz	FOV mm	FA deg	ST mm	Gap mm	ETL	Matrix voxels	Frequency	NEX
**T/R coil 3D SPGR (structural)**	8	3	450	31.25	256	20°	1	0	1	256 x 256	A/P	1
**T/R coil GRE-EPI multiphase (fMRI)**	3010	30	N/A	62.5	240	84°	3	0	1	64 x 64	R/L	1
**body coil 3D SPGR (structural)**	8	3	450	31.25	256	20	1	0	1	256x256	A/P	1
**body coil GRE-EPI multiphase (fMRI)**	2151	30	n/a	62.5	240x 240	76	4	0	1	64 x 64	l/r	1

Supplementary Table 2: MRI acquisition parameters for structural and functional imaging. 3T MRI scanner with a quadrature birdcage transmit-receive (T/R) head coil (GE Model 2376114) or a body-transmit coil (GE 2380637-2) was used. 3D SPGR is a T1-weighted sequence acquired for structural information and GRE-EPI multiphase is used to acquire fMRI data. A/P = anterior/posterior; BW = bandwidth; ETL = echo train length; deg=degree; FA = flip angle; FOV = field of view; GRE-EPI = gradient recalled echo echo planar imaging; Hz = Hertz; L= left; NA = not available; mm=millimeter; NEX = number of excitations; R = right; SPGR = spoiled gradient recalled; ST = slice thickness; TE = echo time; TI = inversion time; TR = repetition time.

Contact Configuration (No. of patients)	Voltage (volts)	Frequency (No. of patients)	Pulse-width (µs)
Monopolar=43<br>Bipolar=11<br>Double monopolar=2<br>Interleaved=1	Optimal=3.5±1.1<br>Suprathreshold=5.2±1.6<br>Subthreshold=2.4±1.1	<100Hz: 13<br>100-150Hz: 32<br>>150Hz: 12	61.5±6.7

Supplementary Table 3: DBS Settings for train and test cohort (N = 57) patients in this study.

Where applicable, numbers of patients or mean ± standard deviation are reported. Source data are provided as a Source Data file. Hz = Hertz, µs = microsecond.

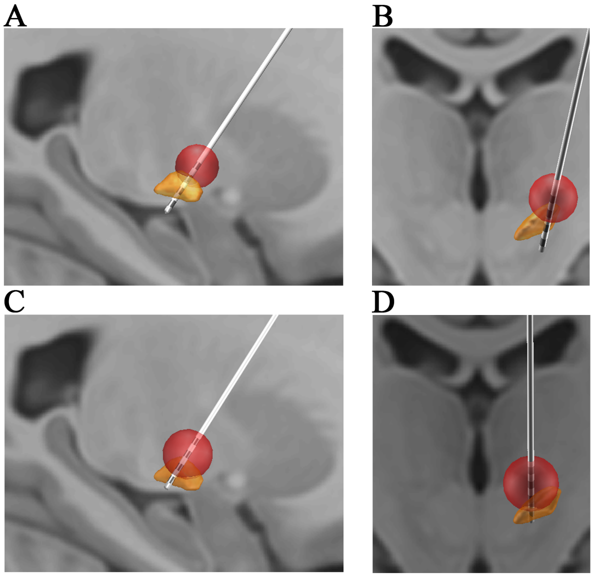

Supplementary Fig. S1: Electrode localization for patients in Figure 3. 3-D reconstruction (Lead-DBS; https://www.lead-dbs.org/) of the electrodes for the contact (A, B) and voltage (C, D) patients in Figure 3. Their volume of tissue activated (shaded red) are overlaid on sagittal (A, C) and coronal (B, D) T-1 weighted standard brain (MNI space ICBM 2009b NLIN asymmetric). The subthalamic nucleus is shown in orange (Ewert et al., 2018). ICBM=International Consortium of Brain Mapping; MNI=Montreal Neurological Institute**.**

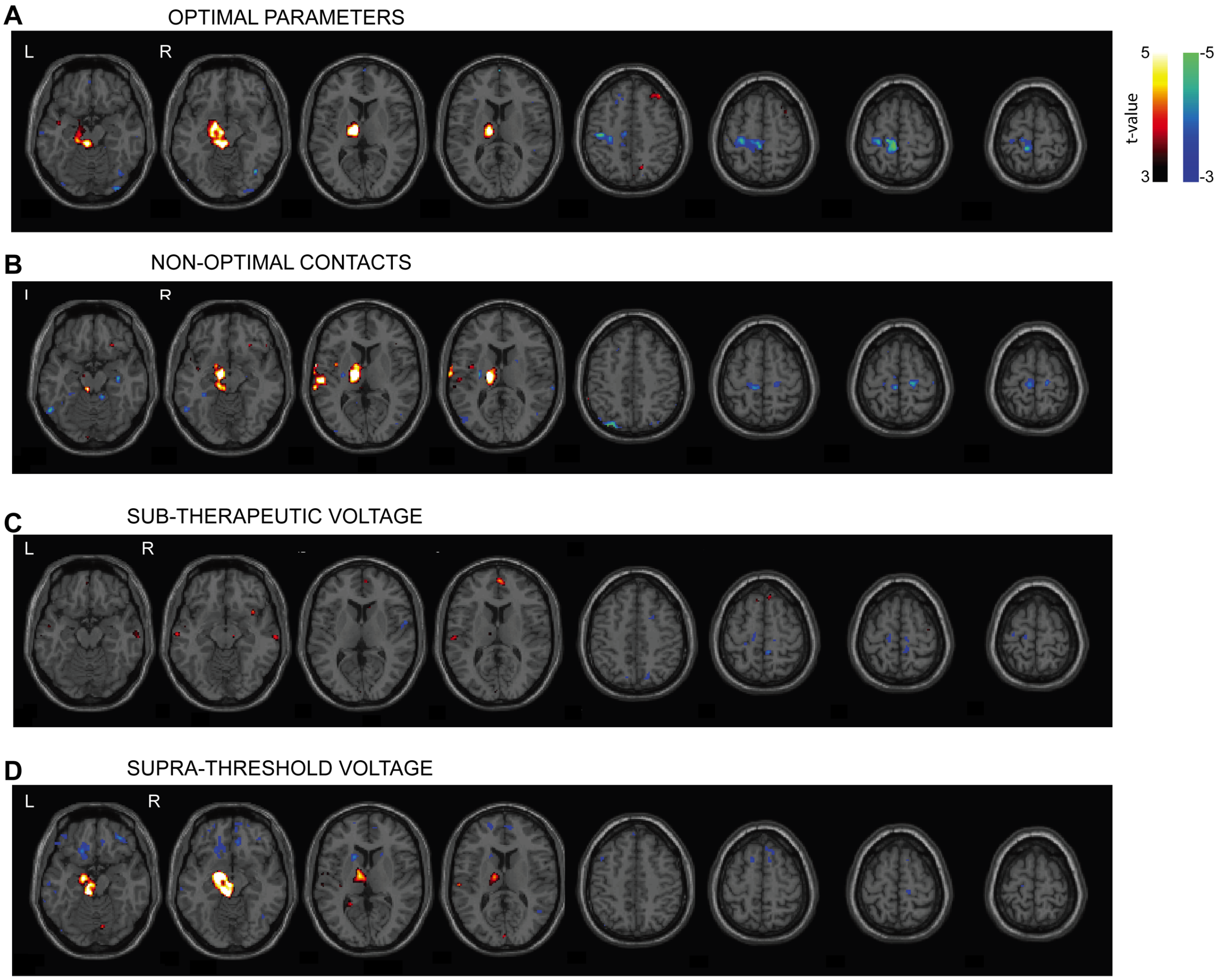

Supplementary Fig. S2: Group analysis of fMRI. (A) Group-level analysis of the fMRI response when the left optimal DBS was turned ON (n=39 total, n=35 STN-DBS and n=4 GPI-DBS, train data) showed significant BOLD changes in motor areas. (B) Non-optimal left DBS contacts (n=50 fMRI sessions from 20 patients, train data) stimulation showed a similar response pattern to (A) but with a reduced magnitude. In addition, fMRI analysis of the non-optimal left contacts showed significant decreased BOLD response in the operculum and visual areas. Brain regions with significant increase (hot colors, positive t-values, DBS-ON>OFF) and decrease (cool colors, negative t-value, DBS-ON<OFF) (p<0·001, cluster size=50) in BOLD response were identified. (C) Non-optimal sub-therapeutic voltages (voltage < optimal voltage) showed responses in the

motor cortex but with reduced amplitude, while (D) supratherapeutic voltages (voltage > optimal voltage) showed activation in non-motor regions. BOLD=blood-oxygen-level-dependent; DBS=deep brain stimulation; fMRI=functional magnetic resonance imaging; GPi=internal globus pallidus; L=left; R=right; STN=subthalamic nucleus.

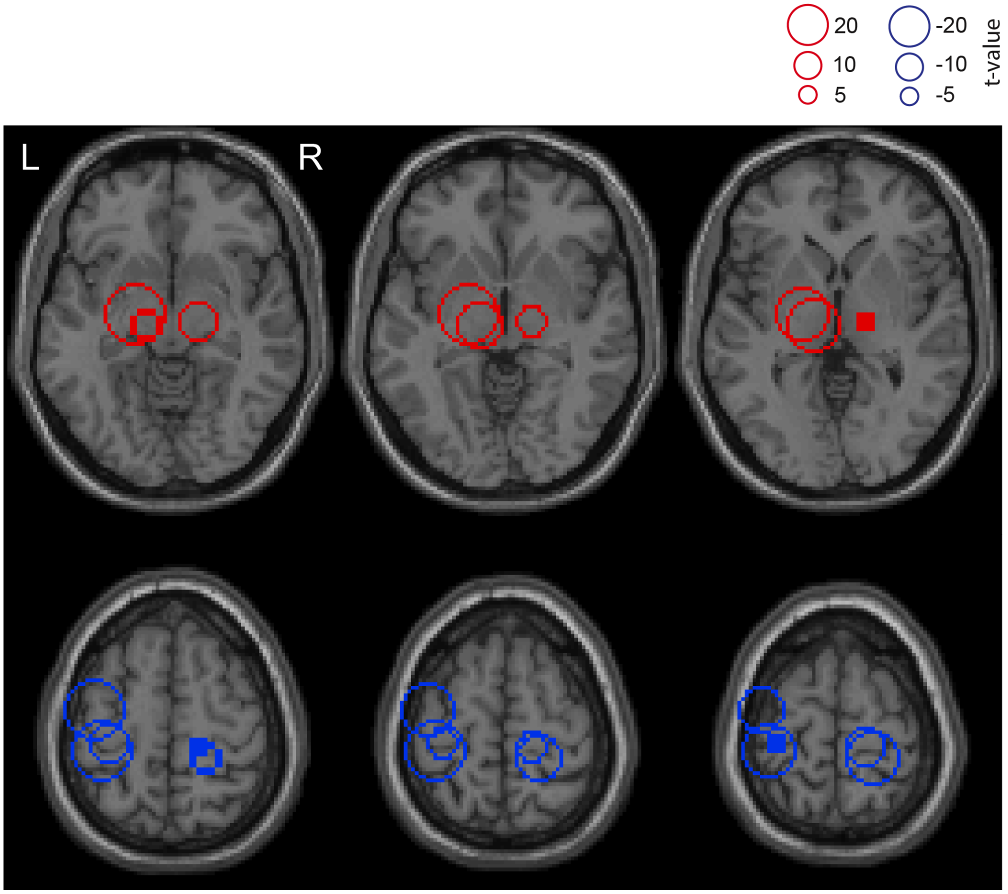

Supplementary Fig. S3: Group analysis of brain fMRI responses to optimal DBS frequency shows a similar response pattern for bilateral stimulation (n=10 patients). The clinically optimized DBS settings of these patients included low (n=4, 60-80Hz) or high (n=6, 150-180 Hz) frequencies in reference to the commonly used 130 Hz (n=10, 5 male, age = 63.1±8.5, UPDRS-III = 39.4 ± 26.5, Levodopa equivalence = 836.36 ± 407.48, days from surgery = 1145±659). Bilateral stimulation was employed in order to mimic programming of frequency, in which bilateral electrodes are commonly evaluated simultaneously. Distribution of peak t-values when the clinically optimal left DBS settings of these patients are used. Voxels corresponding to peak t-value (DBS ON>OFF and DBS ON<OFF) were identified and are shown on a standard Montreal Neurological Institute (MNI) brain. The size of the circle indicates the corresponding t-value. Red circles reflect increased BOLD activity (DBS ON>OFF) whereas blue circles indicate decreased

BOLD activity (DBS ON<OFF). Similar to optimal contacts and voltages, the motor cortex and thalamus demonstrated a change in BOLD signal. BOLD=blood-oxygen-level-dependent; DBS=deep brain stimulation; fMRI=functional magnetic resonance imaging; UPDRS-III=Unified Parkinson's Disease Rating Scale Part III.

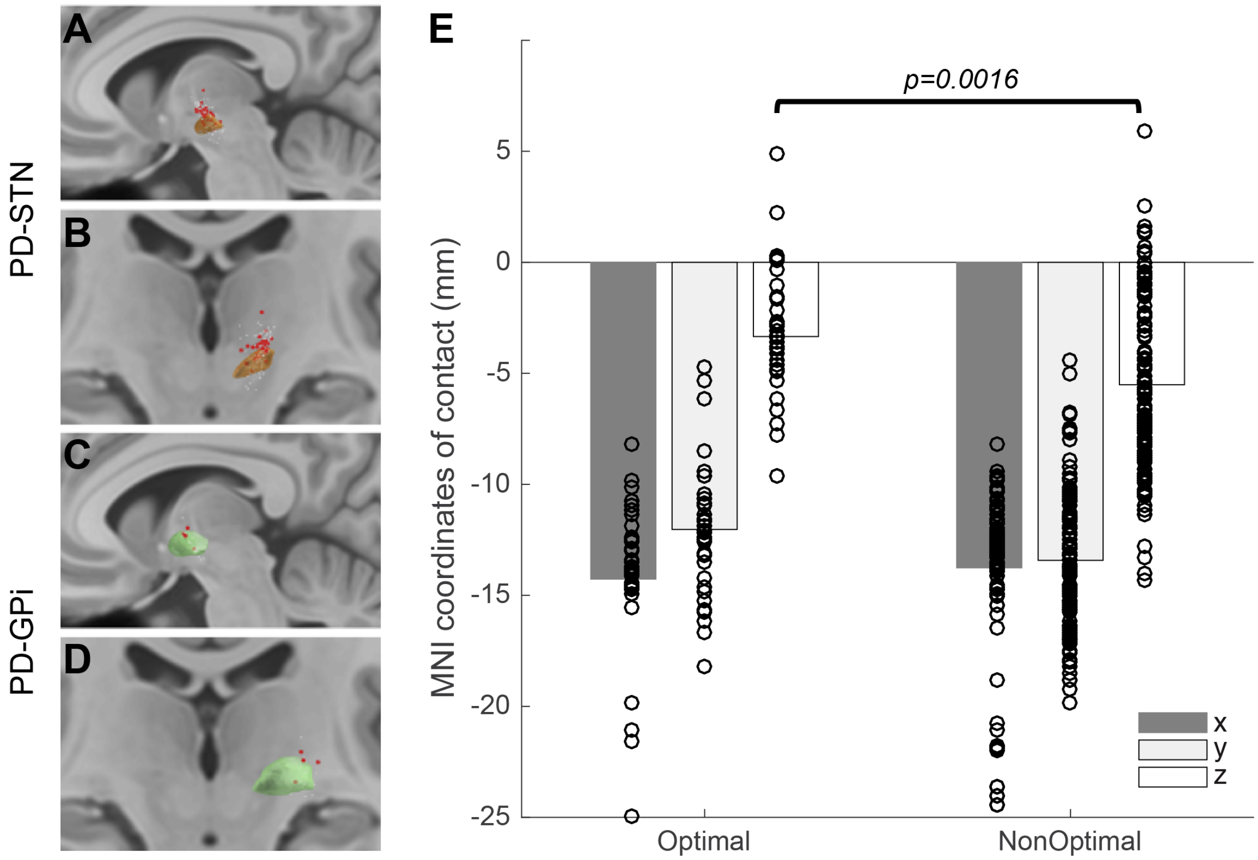

Supplementary Fig. S4: Electrode positions of Optimal and non-optimal contacts in MNI coordinates. Optimal (red) and non-optimal (grey) STN-DBS (A, B) and GPi-DBS (C, D) contacts for the train dataset (n = 39) are overlaid on sagittal (A, C) and coronal (B, D) T1-weighted standard brain (MNI space). The subthalamic nucleus and internal globus pallidus are shown in orange and green, respectively.[17] (E) Mean z-location of the electrode position was significantly different for optimal compared to non-optimal contacts (p=0.0016, two-sided Wilcoxon rank sum test, n=39 optimal and n=117 non-optimal settings from n=39 subjects in the train dataset). Black circles indicate the individual contact locations. Source data are provided as a Source Data file. DBS=deep brain stimulation; GPi=internal globus pallidus; MNI=Montreal Neurological Institute; STN=subthalamic nucleus.

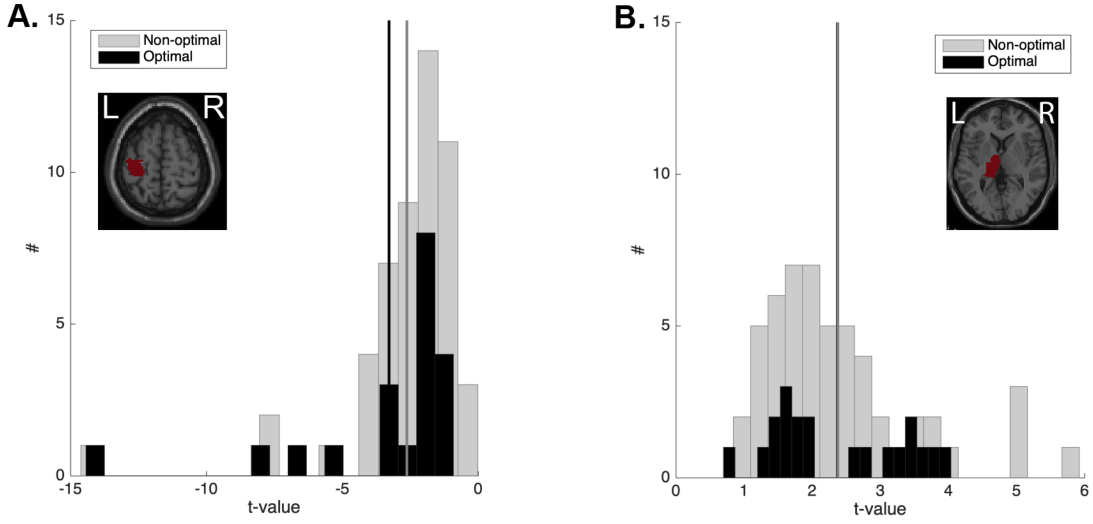

Supplementary Figure S5: Distribution of fMRI activation (t-values, not normalized) for left motor cortex (A) and thalamus (B) across optimal and non-optimal contacts (n=39, train data). The solid lines indicate the mean of the distribution. While left motor cortex showed significant difference between optimal and non-optimal activation, left thalamic activation was similar across the two conditions. Left motor cortex (A) and left thalamus (B) ROI are shown in shaded red. Source data are provided as a Source Data file. fMRI=functional magnetic resonance imaging**.**

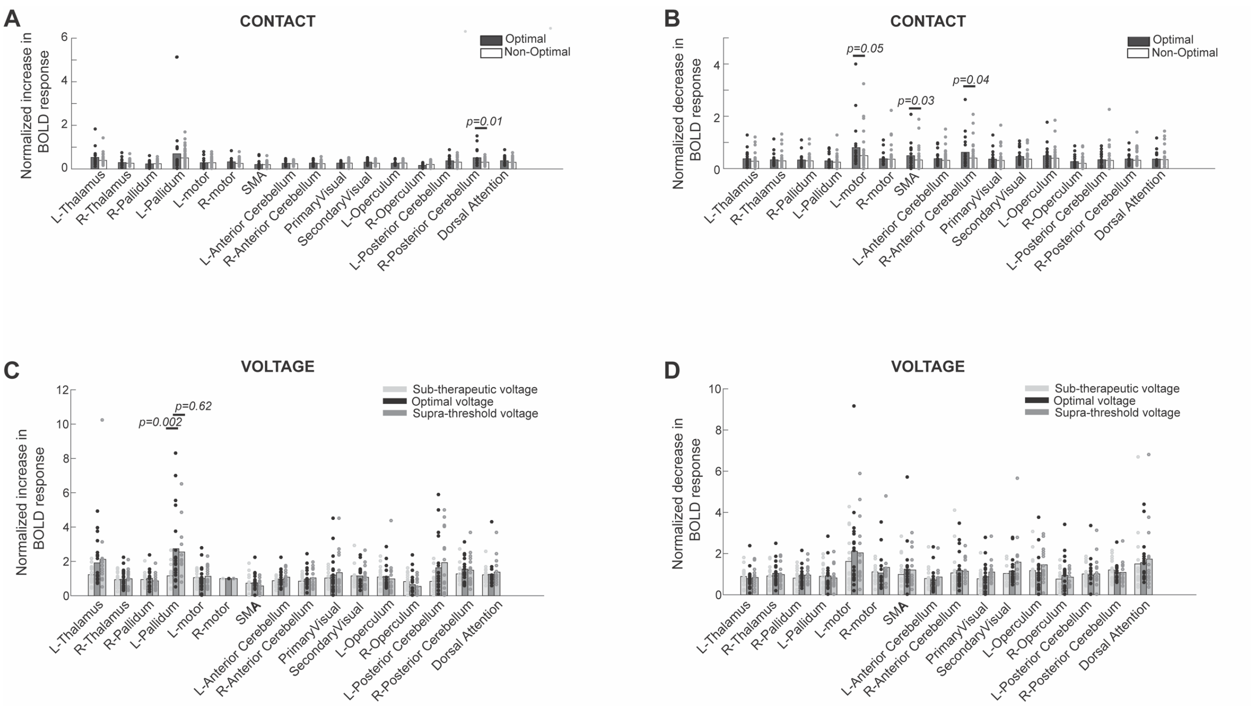

Supplementary Fig. S6: BOLD response of the 16 ROIs included in the analysis (n = 39, train data). (A) Normalized increase and (B) decrease in BOLD fMRI responses for optimal and non-optimal contacts. For each ROI, t-values were normalized by the t-value in the visual and operculum ROIs. Right posterior cerebellum ROIs showed significantly higher BOLD response in optimal compared to non-optimal contact settings. Left motor and right anterior cerebellum ROIs showed significantly greater decreases in BOLD response in the optimal compared to non-optimal contact settings (one-sided Wilcoxon's rank sum test). Data from n=18 optimal and n=52 non-optimal contact settings (n=18 patients, train data contact with at least one non-optimal contact, Table 1). (C) Normalized increase and (D) decrease in BOLD fMRI responses for optimal and non-optimal voltages. Data from n=19 optimal, n=16 non-optimal sub-therapeutic, and n=15 non-optimal supra-therapeutic voltage settings (n=19 subjects, train data voltage, Table 1). For each ROI, t-values were normalized by the t-value in the right motor cortex. Left pallidum ROI showed significantly higher BOLD response in optimal compared to non-optimal voltage settings (two-

sided Wilcoxon rank sum test). Error bars indicate standard error of mean. Source data are provided as a Source Data file. BOLD=blood-oxygen-level-dependent; fMRI=functional magnetic resonance imaging; L=left; R=right; ROI=regions-of-interest; SMA=supplementary motor area.

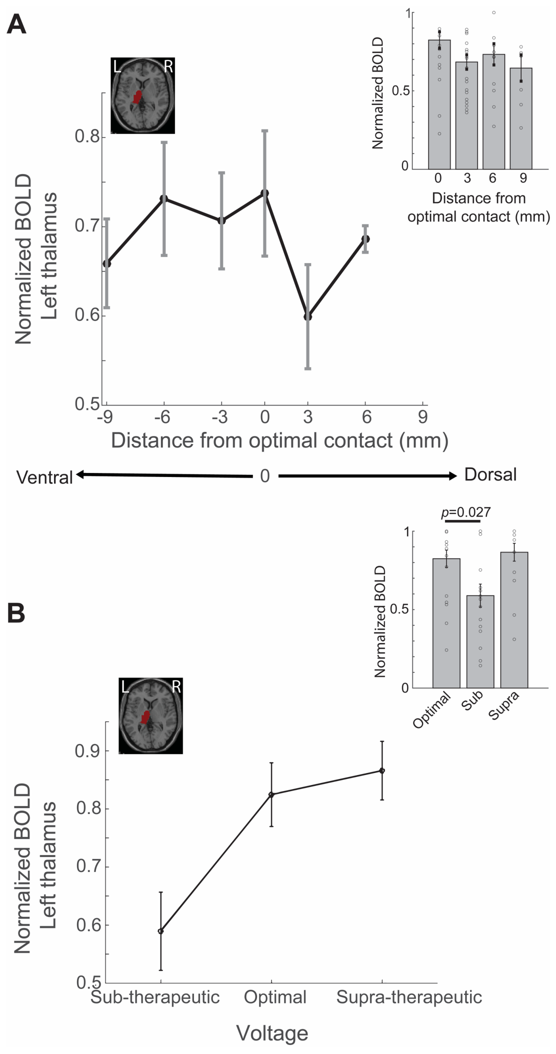

Supplementary Fig. S7: Thalamic BOLD responses associated with optimal versus non-optimal stimulation in DBS patients (n=39, train data). Notations are the same as **Fig. 4B, D**. Mean normalized t-values at the left thalamus are shown as a function of distance to the (A) optimal contact (inset, n=20 (optimal), n=22 (3mm), n=13 (6mm), n=8 (9mm), train data contact with at least one non-optimal contact, Table 1, two-sided Wilcoxon rank sum test) and (B) different voltage settings (n=19 optimal voltage, n=15 supra-therapeutic, and n=16 sub-therapeutic voltage settings, train data voltage, Table 1, two-sided Wilcoxon's rank sum test). The thalamus BOLD signal differentiated only the left optimal from non-optimal subtherapeutic voltage setting. Left thalamus ROI is shown in shaded red. Error bars indicate standard error of mean. Source data are

provided as a Source Data file. BOLD=blood-oxygen-level-dependent; DBS=deep brain stimulation; L=left; R = right; ROI=regions-of-interest.

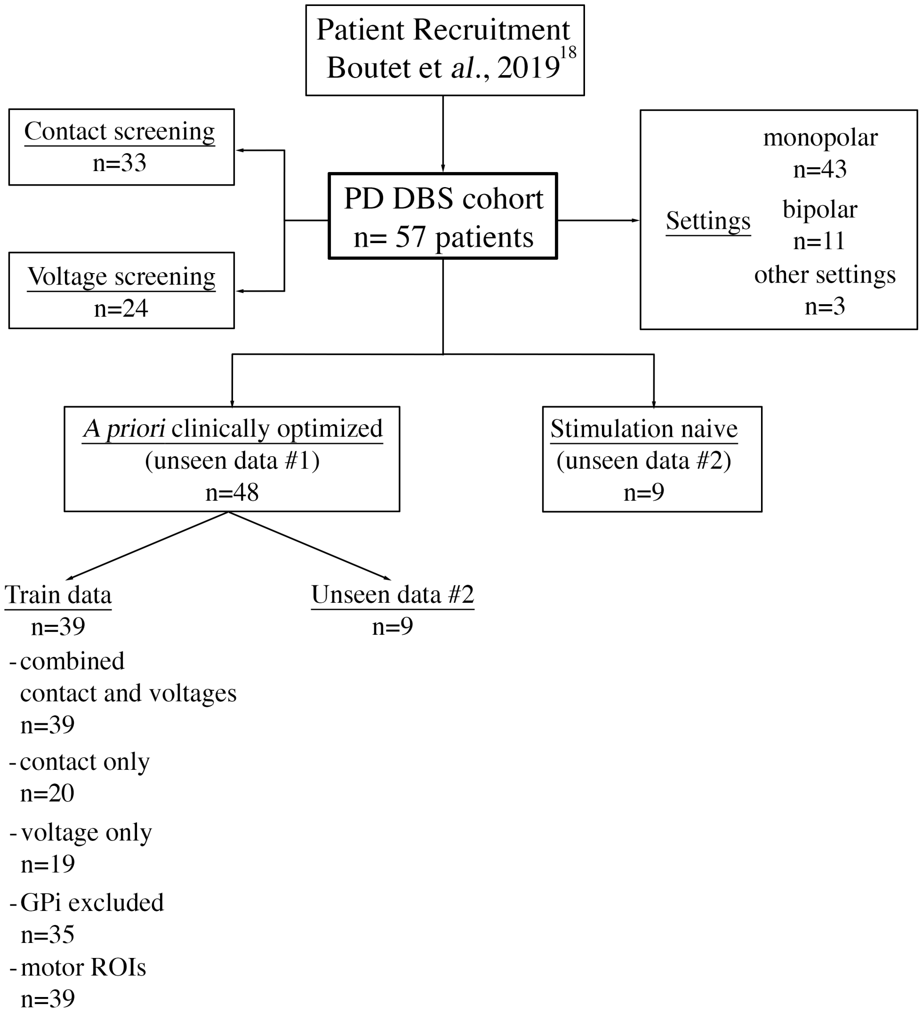

Supplementary Fig. S8: Study flowchart[18]. Division of the PD DBS cohort used in training and testing the machine learning algorithm. DBS=deep brain stimulation; PD=Parkinson's disease. ROI=regions-of-interests.

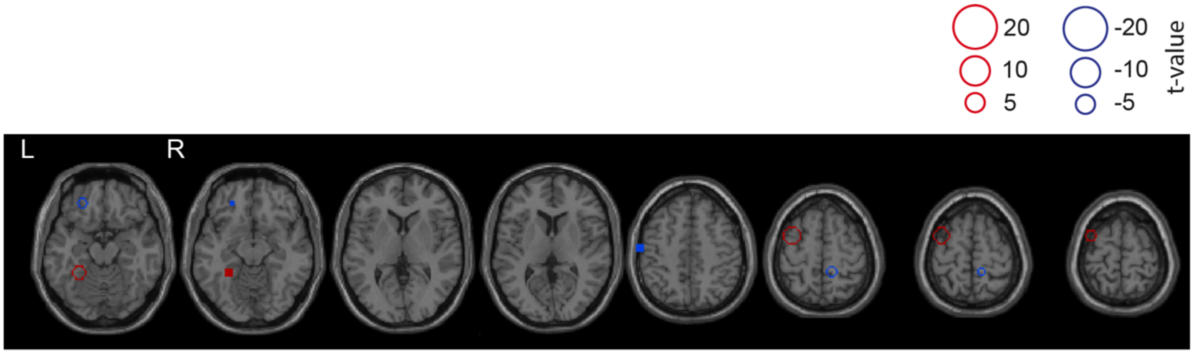

Supplementary Fig. S9: Group analysis of brain fMRI responses to optimal DBS stimulation for GPi-DBS (n=4, train data). Distribution of peak t-values when the clinically optimal left GPi-DBS settings are used (n=4 GPI-DBS, train data). Voxels corresponding to peak t-value (DBS ON>OFF and DBS ON<OFF) were identified and are shown on a standard Montreal Neurological Institute (MNI) brain. The size of the circle indicates the corresponding t-value. Red circles reflect increased BOLD activity (DBS ON>OFF) whereas blue circles indicate decreased BOLD activity (DBS ON<OFF). BOLD=blood-oxygen-level-dependent; DBS=deep brain stimulation; fMRI=functional magnetic resonance imaging; GPi=internal globus pallidus; L=left; MNI=Montreal Neurological Institute; R=right.

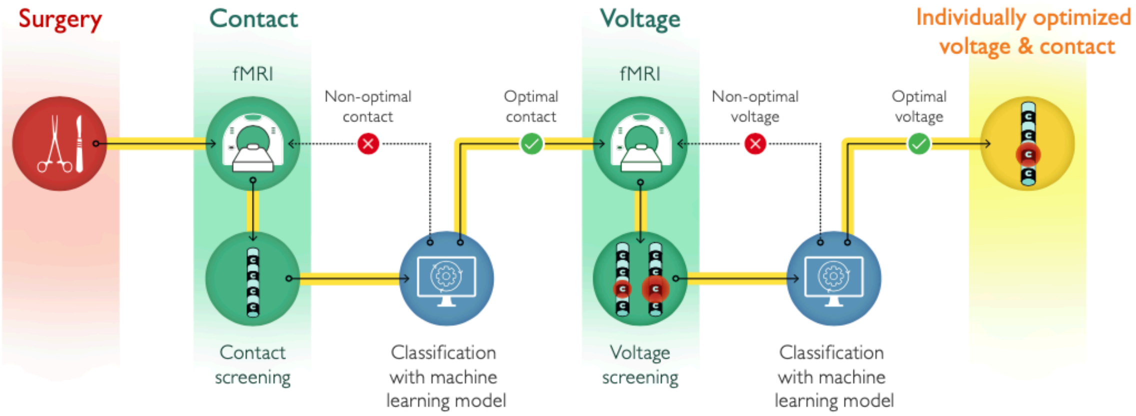

Supplementary Fig. S10: **fMRI-based DBS programming.** Proposed fMRI-based programming workflow based on the results of this study. After DBS surgery, patients would first undergo fMRI screening to identify the optimal electrode contacts followed by voltage adjustments. Future studies may use this workflow as an adjunct to current empirical algorithms to assess various outcomes such as clinical benefits, time to stimulation optimization, number of hospital visits, and cost-effectiveness analysis. DBS=deep brain stimulation; fMRI=functional magnetic resonance imaging.

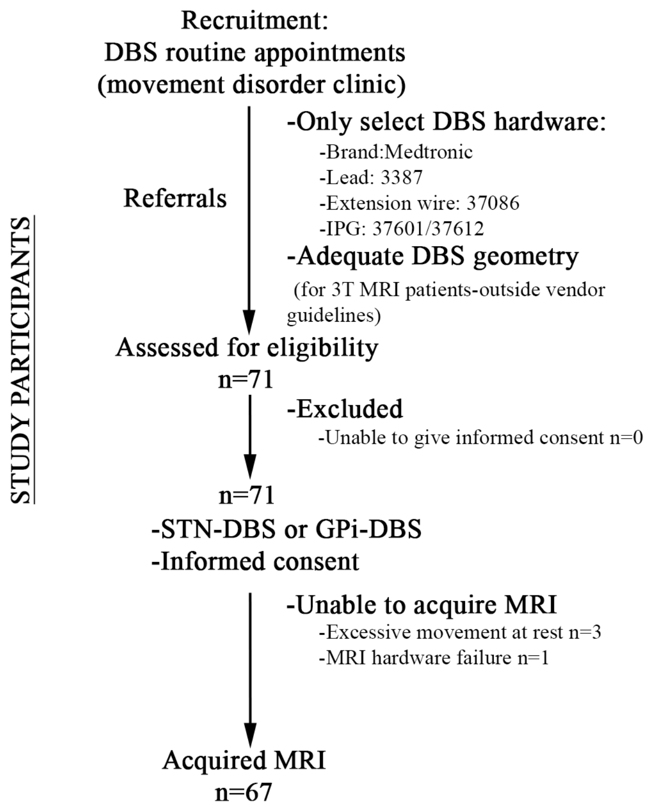

Supplementary Fig. S11: Attrition chart of study participants. DBS=deep brain stimulation; GPi=internal globus pallidus; IPG=internal pulse generator; MRI=magnetic resonance imaging; STN=subthalamic nucleus.

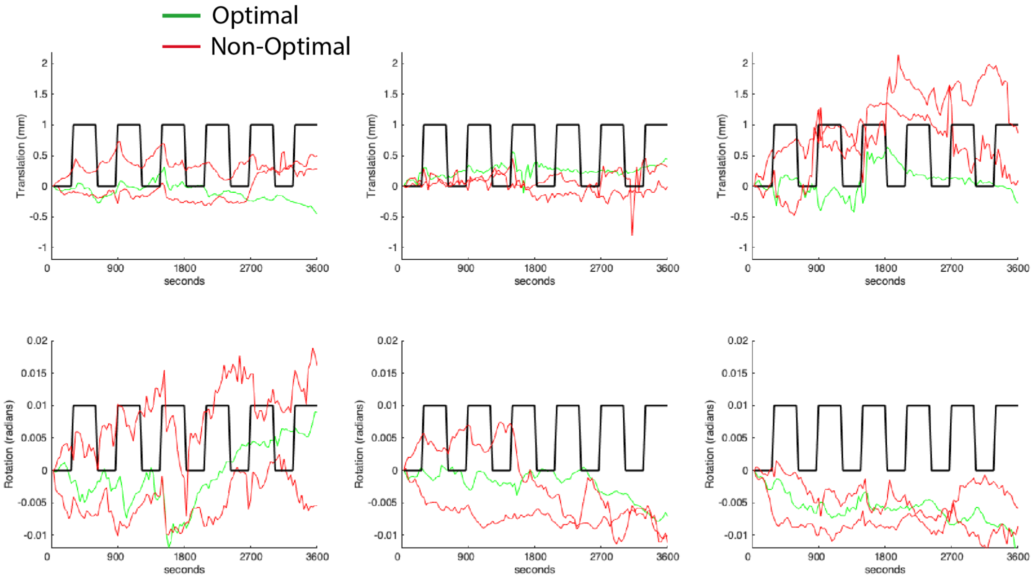

Supplementary Fig. S12: Motion parameters (translation and rotation components) overlaid on DBS ON/OFF block design for the patient shown in Fig. 3A. Motion parameters and DBS block design were uncorrelated (Pearson's correlation coefficient, $r<0.3$) for optimal and non-optimal settings. Each panel represents one of the six motion parameters (3 translation (top row) and 3 rotation parameters (bottom row)). Motion parameters for the optimal and the two non-optimal contacts are shown in green and red, respectively. Source data are provided as a Source Data file. DBS=deep brain stimulation.

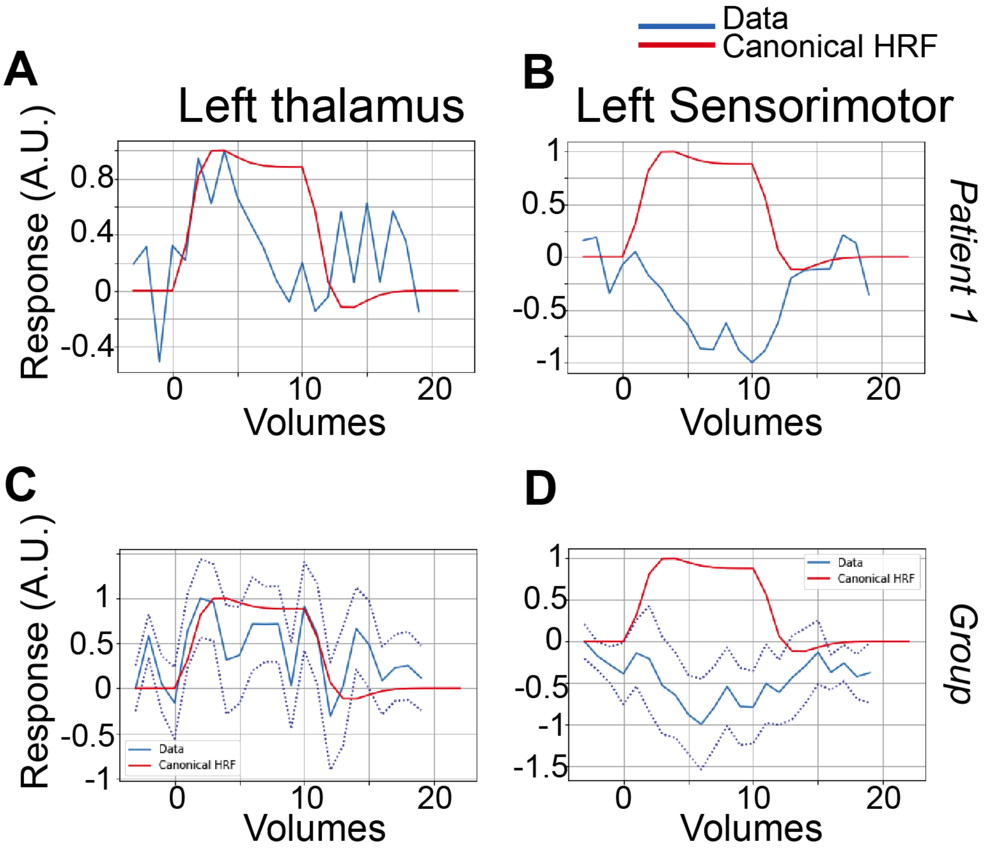

Supplementary Fig. S13: Hemodynamic response function in different ROIs. (A-B) Average time course (blue) for an example PD-STN patient in the (A) left thalamus and (B) left sensorimotor cortex. (D-F) Average time course (solid blue line) across n=20 contact patients (train data) in the (C) left thalamus and (D) left motor cortex. Dotted blue line is the standard deviation time course. Red line shows the canonical double gamma HRF. Y-axis is the fMRI response in arbitrary units (A.U.). Source data are provided as a Source Data file. HRF=hemodynamic response function; ROI=regions-of-interest

References:

1. Horn, A. *et al.* Deep brain stimulation induced normalization of the human functional connectome in Parkinson's disease. *Brain* **142**, 3129–3143 (2019).
2. Gratwicke, J. *et al.* Bilateral Deep Brain Stimulation of the Nucleus Basalis of Meynert for Parkinson Disease Dementia: A Randomized Clinical Trial. *JAMA Neurol.* **75**, 169–178 (2018).
3. Mueller, K. *et al.* Brain connectivity changes when comparing effects of subthalamic deep brain stimulation with levodopa treatment in Parkinson's disease. *NeuroImage Clin.* **19**, 1025–1035 (2018).
4. Holiga, Š. *et al.* Resting-state functional magnetic resonance imaging of the subthalamic microlesion and stimulation effects in Parkinson's disease: Indications of a principal role of the brainstem. *NeuroImage Clin.* **9**, 264–274 (2015).
5. Mueller, K., Jech, R. & Schroeter, M. L. Deep-brain stimulation for Parkinson's disease. *N. Engl. J. Med.* **368**, 482–483 (2013).
6. Saenger, V. M. *et al.* Uncovering the underlying mechanisms and whole-brain dynamics of deep brain stimulation for Parkinson's disease. *Sci. Rep.* **7**, 1–14 (2017).
7. Kahan, J. *et al.* Resting state functional MRI in Parkinson's disease: the impact of deep brain stimulation on 'effective' connectivity. *Brain J. Neurol.* **137**, 1130–1144 (2014).
8. Kahan, J. *et al.* Therapeutic Subthalamic Nucleus Deep Brain Stimulation Reverses Cortico-Thalamic Coupling during Voluntary Movements in Parkinson's Disease. *PLOS ONE* **7**, e50270 (2012).

9. Knight, E. J. *et al.* Motor and Nonmotor Circuitry Activation Induced by Subthalamic Nucleus Deep Brain Stimulation in Patients With Parkinson Disease: Intraoperative Functional Magnetic Resonance Imaging for Deep Brain Stimulation. *Mayo Clin. Proc.* **90**, 773–785 (2015).

10. Jech, R. *et al.* The Subthalamic Microlesion Story in Parkinson's Disease: Electrode Insertion-Related Motor Improvement with Relative Cortico-Subcortical Hypoactivation in fMRI. *PLOS ONE* **7**, e49056 (2012).

11. Phillips, M. D. *et al.* Parkinson disease: pattern of functional MR imaging activation during deep brain stimulation of subthalamic nucleus--initial experience. *Radiology* **239**, 209–216 (2006).

12. Hesselmann, V. *et al.* Intraoperative functional MRI as a new approach to monitor deep brain stimulation in Parkinson's disease. *Eur. Radiol.* **14**, 686–690 (2004).

13. Stefurak, T. *et al.* Deep brain stimulation for Parkinson's disease dissociates mood and motor circuits: a functional MRI case study. *Mov. Disord. Off. J. Mov. Disord. Soc.* **18**, 1508–1516 (2003).

14. Jech, R. *et al.* Functional magnetic resonance imaging during deep brain stimulation: a pilot study in four patients with Parkinson's disease. *Mov. Disord. Off. J. Mov. Disord. Soc.* **16**, 1126–1132 (2001).

15. de Almeida Marcelino, A. L., Horn, A., Krause, P., Kühn, A. A. & Neumann, W.-J. Subthalamic neuromodulation improves short-term motor learning in Parkinson's disease. *Brain* **142**, 2198–2206 (2019).

16. Horn, A. *et al.* Connectivity Predicts deep brain stimulation outcome in Parkinson disease. *Ann. Neurol.* **82**, 67–78 (2017).

17. Ewert, S. *et al.* Toward defining deep brain stimulation targets in MNI space: A subcortical atlas based on multimodal MRI, histology and structural connectivity. *NeuroImage* (2017) doi:10.1016/j.neuroimage.2017.05.015.

18. Boutet, A. *et al.* Functional MRI Safety and Artifacts during Deep Brain Stimulation: Experience in 102 Patients. *Radiology* **293**, 174–183 (2019).
