# Supplementary material for: Predicting optimal deep brain stimulation parameters for Parkinson’s disease using functional MRI and machine learning
Source: Nat Commun. 2021 May 24;12:3043. doi: 10.1038/s41467-021-23311-9 (PMC8144408; doi:10.1038/s41467-021-23311-9)
Supplement: Supplementary file 3 — Reporting Summary [file 41467_2021_23311_MOESM3_ESM.pdf]

nature research

Corresponding author(s): Andres Lozano
Last updated by author(s): 02/08/2021

Reporting Summary

Nature Research wishes to improve the reproducibility of the work that we publish. This form provides structure for consistency and transparency in reporting. For further information on Nature Research policies, see our Editorial Policies and the Editorial Policy Checklist.

Statistics

For all statistical analyses, confirm that the following items are present in the figure legend, table legend, main text, or Methods section.

n/a	Confirmed	
☐	☒	The exact sample size (n) for each experimental group/condition, given as a discrete number and unit of measurement
☒	☐	A statement on whether measurements were taken from distinct samples or whether the same sample was measured repeatedly
☐	☒	The statistical test(s) used AND whether they are one- or two-sided<br>*Only common tests should be described solely by name; describe more complex techniques in the Methods section.*
☒	☐	A description of all covariates tested
☐	☒	A description of any assumptions or corrections, such as tests of normality and adjustment for multiple comparisons
☐	☒	A full description of the statistical parameters including central tendency (e.g. means) or other basic estimates (e.g. regression coefficient) AND variation (e.g. standard deviation) or associated estimates of uncertainty (e.g. confidence intervals)
☐	☒	For null hypothesis testing, the test statistic (e.g. F, t, r) with confidence intervals, effect sizes, degrees of freedom and P value noted<br>*Give P values as exact values whenever suitable.*
☒	☐	For Bayesian analysis, information on the choice of priors and Markov chain Monte Carlo settings
☒	☐	For hierarchical and complex designs, identification of the appropriate level for tests and full reporting of outcomes
☒	☐	Estimates of effect sizes (e.g. Cohen's d, Pearson's r), indicating how they were calculated

Our web collection on statistics for biologists contains articles on many of the points above.

Software and code

Policy information about availability of computer code

Data collection	No software used for data collection.
Data analysis	- MATLAB (MATLAB, The MathWorks, Inc., Version R2017b. Natick, MA, USA)<br>- SPM12 (SPM12; https://www.fil.ion.ucl.ac.uk/spm/software/spm12/)

For manuscripts utilizing custom algorithms or software that are central to the research but not yet described in published literature, software must be made available to editors and reviewers. We strongly encourage code deposition in a community repository (e.g. GitHub). See the Nature Research guidelines for submitting code & software for further information.

Data

Policy information about availability of data

All manuscripts must include a data availability statement. This statement should provide the following information, where applicable:
- Accession codes, unique identifiers, or web links for publicly available datasets
- A list of figures that have associated raw data
- A description of any restrictions on data availability

The data and code that support the central findings of this study are available from the corresponding author.

Field-specific reporting

Life sciences study design

All studies must disclose on these points even when the disclosure is negative.

Sample size	The study was exploratory in nature and deals with a heterogeneous patient population. As such, we did not use a prespecified sample size calculation. Based on our preliminary data, there were striking changes in the fMRI pattern with DBS-ON vs DBS-OFF at the individual and group levels. Preliminary results showed that approximately 12 patients were required to build a preliminary successful machine learning algorithm for contact settings. Our total cohort of patients in which fMRI was acquired includes 67 patients (203 fMRI sessions), which should be sufficient to generate meaningful findings.
Data exclusions	No data was excluded, however fMRI data could not be acquired in 4 patients due to excessive movement or MRI hadrware failure.
Replication	We built a machine learning model to predict optimal vs non-optimal DBS settings. We obtained a train data accuracy (5-fold cross validation framework). The validity of the classifier model was assessed by testing it on two groups of unseen, independent data sets: 9 patients a priori clinically optimized by the neurologist and 9 stimulation-naïve patients prior to initiation of programming. Each patient's fMRI response maps were fed to the ML model to prospectively predict the optimal DBS setting. Further models were trained with different patients' subsets: without GPi and including only sub-cohorts of contact-only or voltage-only and the results are reported.
Randomization	fMRI were acquired prospectively with the intent of building a machine learning model predicting clinically optimized DBS settings. The order in which non-optimal contact or voltage stimulation was delivered was randomized. Each subject was randomly assigned to one of train or test cohort for the machine learning experiments.
Blinding	58/67 patients in which fMRI was acquired had been receiving chronic DBS stimulation and their stimulation at the time of the fMRI (or the settings at 1 year after the surgery for those who underwent the fMRI before 1-year post-op) were deemed clinically optimized. Clinically optimal DBS settings for these patients were obtained using published algorithms. 9 patients were stimulation-naïve patients who recently (<1 month of programming) underwent DBS surgery (i.e., no clinically defined optimized DBS settings at the time of the MRI). They received clinical programming by a neurologist blinded to the fMRI results. Two movement disorder neurologists, who previously published programming algorithms, were involved in the optimization of the patients. During the fMRI, the patients were blinded to the DBS settings.

Reporting for specific materials, systems and methods

We require information from authors about some types of materials, experimental systems and methods used in many studies. Here, indicate whether each material, system or method listed is relevant to your study. If you are not sure if a list item applies to your research, read the appropriate section before selecting a response.

Materials & experimental systems

n/a	Involved in the study	
☒	☐	Antibodies
☒	☐	Eukaryotic cell lines
☒	☐	Palaeontology and archaeology
☒	☐	Animals and other organisms
☐	☒	Human research participants
☐	☒	Clinical data
☒	☐	Dual use research of concern

Methods

n/a	Involved in the study	
☒	☐	ChIP-seq
☒	☐	Flow cytometry
☐	☒	MRI-based neuroimaging

Human research participants

Policy information about studies involving human research participants

Population characteristics	fMRI data was acquired in 67 Parkinson's disease patients receiving DBS (203 fMRI sessions). The mean age was 62.9 years ±8. There were 41 females and 26 males.
Recruitment	Patients were recruited during their DBS routine clinical appointments. Inclusion criteria were (1) participants receiving active STN- or GPi-DBS, (2) ability to provide written informed consent, and (3) specific models of Medtronic DBS hardware, including DBS leads (3387, 28 cm; Medtronic, Minneapolis, MN), extension wire (37086, 60cm; Medtronic, Minneapolis, MN) and IPG (Activa PC 37601, Activa RC 37612, Medtronic, Minneapolis, MN). Participants undergoing 3T MRI were also required to have DBS hardware geometry similar to previous phantoms. While we recruited all patients within these inclusion criteriae, patients were invited to volunteer for the study and it is plausible that they may have displayed similar characteristics, for example in terms of personality and inclication to participate in trials.
Ethics oversight	University Health Network Research Ethics Board (ID: #14-8255).

Note that full information on the approval of the study protocol must also be provided in the manuscript.

Clinical data

Policy information about clinical studies

All manuscripts should comply with the ICMJEguidelines for publication of clinical research and a completedCONSORT checklist must be included with all submissions.

Clinical trial registration	NCT03153670
Study protocol	Study protocols are available from clinicaltrials.gov website and the corresponding author by request.
Data collection	MRI data was acquired at Toronto Western Hospital (Toronto, Ontario, Canada) from June 2017 to February 2020. The associated clinical data was acquired at the Movement Disorder Clinic at Toronto Western Hospital (Toronto, Ontario, Canada) during the same time period.
Outcomes	The primary outcome was brain areas engaged with deep brain stimulation as assessed by changes in BOLD signal on functional magnetic resonance imaging (fMRI) images.

Magnetic resonance imaging

Experimental design

Design type	6.5-minute fMRI sessions acquired using a 30s DBS-ON/OFF cycling paradigm (block design).
Design specifications	30-second block; each session was 6.5 minutes (13 blocks).
Behavioral performance measures	DBS settings (contacts or voltages) were categorized as optimal or non-optimal by a movement disorder neurologist using published algorithms.

Acquisition

Imaging type(s)	Structural and functional MRI
Field strength	3T
Sequence & imaging parameters	T/R coil: 3D SPGR TR=8ms, TE=3ms, FOV=256mm, Matrix=256x256 and GRE-EPI (fMRI) TR=3010ms, TE=30ms, FOV=240mm, Matrix=64x64.<br>body-coil: 3D SPGR TR=8ms, TE=3ms, FOV=256mm, Matrix=256x256 and GRE-EPI (fMRI) TR=2151ms, TE=30ms, FOV=240mm, Matrix=64x64.
Area of acquisition	whole brain scan

Diffusion MRI ☐ Used ☒ Not used

Preprocessing

Preprocessing software	All fMRI data were slice time corrected, motion corrected, rigidly registered to a T1-weighted image, non-linearly registered to a standard space MNI brain, and spatially smoothed using a FWHM 6mm gaussian kernel in SPM12 (http://www.fil.ion.ucl.ac.uk).
Normalization	All fMRI data were non-linearly registered to a standard space MNI brain, and spatially smoothed using a FWHM 6mm gaussian kernel in SPM12 (http://www.fil.ion.ucl.ac.uk).
Normalization template	Standard space MNI brain provided in SPM12 (http://www.fil.ion.ucl.ac.uk).
Noise and artifact removal	All fMRI data were motion corrected (6 parameters) in SPM12 (http://www.fil.ion.ucl.ac.uk). The estimated parameters for 6-degrees of motion were used as regressors to the design matrix used for calculating statistical parametric maps.
Volume censoring	To account for artifacts due to head motion in PD patients, we used the Art toolbox to detect and remove volumes with motion >1.5mm. Overall, for any given patient, this resulted in the removal of a maximum of 6 volumes (3.3%) from the total volumes acquired.

Statistical modeling & inference

Model type and settings	Individual fMRI statistical parametric maps (functional response t-maps) were estimated using a 30 second DBS-ON/OFF block design with the canonical double gamma function for modeling the hemodynamic response function (HRF). Functional response maps were corrected for multiple comparisons using a p-value of 0.001, with cluster level thresholding of 50 voxels, to give an overall p-value of less than 0.05 for visualization. While the cluster threshold was used for visualization purposes, the machine learning model was constructed using unthresholded t-values to retain the full spectrum of the data. We used 16 ROIS and a linear-discriminant analysis (LDA) within a 5-fold cross validation framework to classify optimal vs non optimal fMRI maps.
Effect(s) tested	For each individual fMRI session, DBS-ON was compared to DBS-OFF. Those fMRI patterns were categorized as clinically

optimal or non-optimal by a movement disorder neurologist using published algorithms. We tested the accuracy of the machine learning model to predict optimal vs non-optimal DBS settings.

Specify type of analysis: ☐ Whole brain ☒ ROI-based ☐ Both

Anatomical location(s): To perform a region-based analysis for each patient, average t-values were determined for 16 ROIs derived from a functional atlas and our experience with DBS fMRI. ROIs included regions in the thalamic-motor circuit such as the thalamus, pallidum, primary motor cortex, anterior cerebellum, and supplementary motor area. Additionally, ROIs from other areas that could be related to common adverse effects (e.g., speech and gait disturbances) observed in PD-DBS patients at non-optimal contacts and voltages during our MRI sessions were included in the analysis. As a result, primary and secondary visual cortex, operculum, and posterior cerebellum were also included.

Statistic type for inference
(See Eklund et al. 2016)

Cluster threshold were only used for visualization purposes. The machine learning model used the full spectrum of the data.

Correction

Functional response maps were corrected for multiple comparisons using a p-value of 0.001, with cluster level thresholding of 50 voxels, to give an overall p-value of less than 0.05 for visualization. While the cluster threshold was used for visualization purposes, the machine learning model was constructed using unthresholded t-values to retain the full spectrum of the data. We used 16 ROIS and a linear-discriminant analysis (LDA) within a 5-fold cross validation framework to classify optimal vs non optimal fMRI maps.

Models & analysis

n/a	Involved in the study
☒	☐ Functional and/or effective connectivity
☒	☐ Graph analysis
☐	☒ Multivariate modeling or predictive analysis

Multivariate modeling and predictive analysis

T-values extracted from 16 ROIs (32 features for each patient) were used to classify the optimal and non-optimal contacts and voltages using a linear-discriminant analysis (LDA) within a 5-fold cross validation framework.
